# Molecular Characterization of MHC Class I Genes in Four Species of the *Turdidae* Family to Assess Genetic Diversity and Selection

**DOI:** 10.1155/2021/5585687

**Published:** 2021-04-10

**Authors:** Muhammad Usman Ghani, Li Bo, An Buyang, Xu Yanchun, Shakeel Hussain, Muhammad Yasir

**Affiliations:** ^1^College of Wildlife Resources and Protected Area, Northeast Forestry University, Harbin 150040, China; ^2^Department of Stem Cell Biology and Medicine, Graduate School of Medical Science, Kyushu University, Fukuoka 810-0000, Japan; ^3^Department of Life Science and Technology, Huazhong Agricultural University, Wuhan, China

## Abstract

In vertebrate animals, the molecules encoded by major histocompatibility complex (MHC) genes play an essential role in the adaptive immunity. MHC class I deals with intracellular pathogens (virus) in birds. MHC class I diversity depends on the consequence of local and global environment selective pressure and gene flow. Here, we evaluated the MHC class I gene in four species of the *Turdidae* family from a broad geographical area of northeast China. We isolated 77 MHC class I sequences, including 47 putatively functional sequences and 30 pseudosequences from 80 individuals. Using the method based on analysis of cloned amplicons (*n* = 25) for each species, we found two and seven MHC I sequences per individual indicating more than one MHC I locus identified in all sampled species. Results revealed an overall elevated genetic diversity at MHC class I, evidence of different selection patterns among the domains of PBR and non-PBR. Alleles are found to be divergent with overall polymorphic sites per species ranging between 58 and 70 (out of 291 sites). Moreover, transspecies alleles were evident due to convergent evolution or recent speciation for the genus. Phylogenetic relationships among MHC I show an intermingling of alleles clustering among the *Turdidae* family rather than between other passerines. Pronounced MHC I gene diversity is essential for the existence of species. Our study signifies a valuable tool for the characterization of evolutionary relevant difference across a population of birds with high conservational concerns.

## 1. Introduction

The major histocompatibility complex (MHC) is a group of molecules encoded by certain genes that are most polymorphic to have been described in vertebrates' genomes [1]. Two types of MHC gene families, class I and class II, are useful to cell surface glycoproteins that regulate the immune response. MHC class II molecules are heterodimers consisting of an *α* chain and a *β* chain; both contribute to presenting peptides from the processing of extracellular pathogens such as bacteria to the CD4+ T-helper cells [2]. Heterodimer molecules of MHC class I are made up of an *α* chain and a non-MHC molecule, the *β*2 microglobulin. The *α* chain constitutes a cytoplasmic tail, a transmembrane domain, and three extracellular domains named *α*1, *α*2, and *α*3 [3] that are encoded by exons 2, 3, and 4, respectively. The MHC class I molecules are expressed in almost all somatic cells and trigger an adaptive immune response by presenting endogenously derived peptides of viral protein and an individual's own body cells to CD8+ cytotoxic T-cells [4]. Polymorphism is largely confined within the region encoding the ABS (antigen-binding site) of MHC class I [5]. Maintenance of surprising diversity is supposed to take place by two types of selection: heterozygote advantage and frequency-dependent selection. Heterozygotes could recognize a broader range of antigens from multiple pathogens and therefore have more fitness than either individual having a homozygote [6]. Other is frequency-dependent selection, in which rare alleles deliver a selective advantage where pathogens have found a means to escape against common immune defensive alleles in the population. Thus, alteration in the pathogen community with time and locality results in MHC variation in the host population. Generally, in an individual possessing huge numbers and diverse MHC alleles; more pathogens can be recognized [1].

Structural diversity and immune response have been explored in numerous research, including genomics [7, 8], ailment [9–11], and mate choice [12–14]. Sequence similarity at PBR-based assignment to the locus is frequently hampered by various evolutionary indicators due to current recombination, duplication, and/or concerted evolution as well as positive selection mediated by a variety of pathogens [15]. Thus, numerous studies emphasized MHC genes as important markers to evaluate the adaptive potential and evolutionary status of a threatened population [16].

The emerging scenario inspires researchers to collect statistics from a group of wild taxa to enlarge our understanding of the evolution of the MHC gene [17]. Despite significant efforts, protocols for locus-specific MHC genotyping in avian are still difficult to achieve and remarkably rare [18]. MHC studies in population of wild birds remain neglected possibly due to complications in amplifying gene sequences from bird species not closely related to systematically studied chicken [19, 20].

A significant decline in habitats and fragmentation of available habitats are predisposing factors for dramatic deterioration in population sizes [21]. The avian genus *Turdus* is one of the broadly distributed passerine genera, with 65 documented extant species. The genus is listed wild territorial birds that are beneficial to china having economic and research value. Birds of this genus are strongly migratory thus experiencing a variety of environments. Up to the present, there are no studies on MHC class I genes in *Turdidae* species, which is the first step towards exploring the role of selection mediated by pathogens in the maintenance of MHC class I diversity. Precisely, this study aims to (1) Measured locus-specific variation in MHC I exon 3 genes across the *Turdidae* family to evaluate the mode of evolution by which such variation comes about. To achieve this, we have measured the diversity and selection at MHC I genes to make available the variations that exist across the *Turdidae* family. (2) We investigate the numbers of alleles possessed by each species and the general features of alleles in terms of functional genetic diversity. (3) Phylogenetic analyses to assess evolutionary relationships and processes driving avian MHC I diversity among four species of the *Turdidae* family and other avian species.

## 2. Material and Methods

### 2.1. Study Population

**Figure 1:**
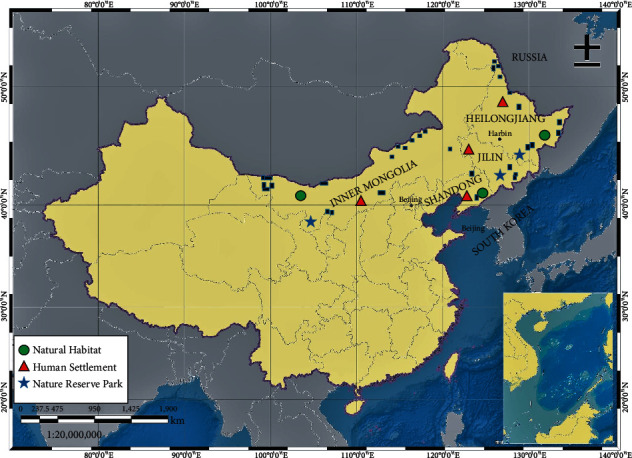
Geographical locations of samples included in our study. Square represents the actual site of the sample, and size of the square represents the approximate diameter of the sample's geographical range.

The study population was non-sympatrically distributed 80 individuals of four species of genus *Turdus* of the *Turdidae* family. Samples include two to three contour feathers, tissue from breast and liver of birds accidentally injured or died during migratory season of 2017-19 in autumn and deposit in State key laboratory of wildlife detection center in northeast forestry university, stored at 4°C. The geographical location of sample material is presented (Figure 1).

### 2.2. Extraction of Genomic DNA

Region of calamus to the rachis of contour feathers was excised, tissues from skeletal muscles were minced, placed into a 1.5 ml Eppendorf tube containing TNE buffer (10 mM Tris-HCl (pH 8.0), 150 mM NaCl, 2 mM EDTA, 1% SDS). Total genomic DNA was extracted with AxyPrep Multisource Genomic DNA Miniprep Kit (AXYGEN, China) according to the manufacturer's instructions. The DNA concentration was measured with Nanopore Spectrophotometer at 260 nm absorbance. Samples above 100 ng/*μ*l concentration were used for further analysis.

### 2.3. PCR, Cloning, and Sequencing

Polymerase chain reaction was conducted using motif specific primers designed for the amplification of MHC class I genes in great reed warbler. The forward primers HN36 5′-TCCCCACAGGTCTCCACACAGT-3′ and HN46 reverse 5′-ATCCCAAATTCCCACCCACCTT-3′ correspond to exon 3 region in the flanking introns, the region coding most of the peptide-binding site in MHC molecules (subunit *α*2) [22–24]. The primers were used due to their successful amplification in many passerine species. Amplification was performed in the reaction mixture containing 20 ng DNA template, 0.2 *μ*M of each primer, 25 *μ*l 2× EasyTaq® PCR SuperMix (+dye) (Trans, China), and water (deionized) to reach 50 *μ*l as final volume. Thermal cycling for MHC class I amplification began with one cycle at 94°C for 5 min, followed by 30 cycles of denaturation consisting of sequential steps of 94°C for 30s, 52°C for 30s, and 72°C for 30s, ending with a single extension step at 72°C for 5 min. Purification was carried out with AxyPrep™DNA Gel Extraction Kit in accordance with the manufacturer's protocol. Purified PCR product was cloned using pEASY ®-T5 Zero Cloning Kit containing Trans1-T1 Phage resistant chemically competent cells (Transgen Biotech). PCRs were performed for positive clones using M13 forward and reverse primers. Several colonies (20-25) per individual were selected and used as a template for sequencing directionally on an automatic sequencer (ABI PRISM 3730; Invitrogen Biotechnology Co. Ltd.).

### 2.4. Definition of Allele

Since few artifacts introduced during the recombination of PCR products in cloning [25, 26]. Amplification, cloning and sequencing were performed twice. Sequences were verified and referred to as an Allele; either minimum of three sequences have the same nucleotide composition or repeated in both events. The sequences which showed any deletion, insertion, or premature stop codons within exons were identified as presumed pseudogene sequence, and others were considered as putative functional allele (PFA) [27]. All sequences appropriate to our criteria have been deposited into the GenBank (Accession No: MN849308-54).

### 2.5. Data Analysis

#### 2.5.1. Sequence Analysis

Chromatogram signals of all sequencing were examined with chromas 2.2.6. Sequences without ambiguous signals were selected. Vector sequence from the MHC class I gene was removed using seqMan in the DNAStar7.1 package. Sequence editing and organization were done with BioEdit [28]. Sequences were aligned individually and then altogether four sampled species using CLUSTAL X [29]. The unique alleles were named according to the nomenclature for MHC in non-human species [30]. NCBI BLAST [31] was used for sequences confirmation representing close identity to passerine species previously published MHC class I exon 3 sequences. Sequences having at least one stop codon (shift in the reading frame due to indels or nonsense sequences) were classified as pseudogenes. Based upon sequences found to be translatable, a minimum number of functional loci MHC class I was estimated using a conservational approach that all Loci from samples species' individual were in heterozygote state.

The average pairwise nucleotide distances (Kimura 2-parameter model - K2P), and the Poisson-corrected amino acid distances were calculated using MEGA7.0. Standard errors were obtained through 1000 bootstrap replicates. Haplotypes identification (*Na)*, the average number of nucleotide differences *(K),* polymorphic sites *(S)*) and nucleotide diversity (*π*) were measured by DnaSP 5.10 [32].

### 2.6. Inference of Recombination

Recombination can influence the outcomes of selection, we first tested recombination. Analyses were implemented for the nucleotide alignment of exon 3 in the Recombination Detection Program version 4 (RDP4). Several method, including RDP [33], GENECONV [34], Chimaera [35], MaxChi [36], BootScan [33], SiScan [37], and 3Seq [38], were used to detect recombination events. In addition, the online GARD tool, provided by the Datamonkey webserver (http://www.datamonkey.org/), was used for recombination signals assessment [39].

### 2.7. Tests for Selection

For selection, we conduct a priori classification of peptide binding region (PBR) and non- peptide region upon inferred passerine PBR sequences [40, 41] homology sites with chicken MHC [42, 43] and human HLA [44]. The identification of sites subjected to selection in MHC class I Exon 3 was performed using various methods. The first standard selection test (Tajima's *D*, Fu and Li's *F*∗, and Fu and Li's *D*∗) were calculated using DnaSP 5.0 [32]. Second method was the calculation of parameter (*ω*) for functional alleles. It was carried out an overall estimation of *d*_N_/*d*_S_ of MHC class I Exon 3 and the other was codons comprising only PBR and non-PBR, which was calculated with MEGA 7.0 according to the Nei-Gojobori method [45] with the Jukes and Cantor correction. Standard error estimates were derived from 1000 bootstrap replicates. *Z* test of historical positive selection [46] was calculated in MEGA 7.0. Third, the Maximum likelihood implemented in codeml in PAML 4.9 was used for identification of sites involved in the positive selection, which are indicated where the ratio *ω* (*d*_N_/*d*_*S*_) larger than 1 [47]. Two different models corresponding *ω* were tested: M7 (beta), M8 (*β* and *ω*). To find whether the alternative model (M8) provided better fitter than the M7, we performed Likelihood ratio tests to compare twice the difference of the log-likelihood ratios (2ΔlnL) using a distribution *χ*^2^. PSSs in the M8 model was identified by PP more than 95% using the Bayes empirical Bayes procedure. Positively selected sites were verified at each codon site separately using many complementary approaches implemented in Datamonkey (http://www.datamonkey.org/) [48] in addition to afore mention methods. Specifically, we used MEME [49], FEL, SALC [50], and FUBER [51].

### 2.8. Phylogenetic Analysis

To assess the phylogenetic relationship, we construct two phylogenies (One for sampled species and other representing MHC class I sequences of related passerines plus sampled species) using Bayesian inference. We find the GTR + T nucleotide substitution model [52] that fits our data using MrModeltest [35] through the Akaike Information Criterion (AICc) [53]. Bayesian Markov chain Monte Carlo (MCMC) was run for two million generations and sampling every 1,000 generations to ascertain when log Likelihood reached stationary phase. The phylogenetic tree was summarized in MrBayes v3.1.2 [54] and the first 25% of the tree as burn-in was removed. Fig tree was used for visualization of the consensus tree. Exploration of relation between sampled species and related avian species, we conducted a maximum likelihood (ML) analysis with MEGA 7.0 [55]. The data were analyzed with the T92 + G model. We conducted 1000 bootstrap replicates to estimate the support. Values greater than 75% were indicated in the ML phylogenetic trees. The species covered are mainly from *Passeridae*, *Acrocephalidae*, *Paridae*, *Motacillidae*, *Muscicapidae*, *Hirundinidae*, *Phylloscopidae*, *Fringillidae*, *Cardinalidae*, and *Sturnidae*. To further identify allelic lineages among sampled species and related avian species, we conducted the Neighbor-Net algorithm in SplitsTree 4.14.8. Neighbor-Net networks were based on uncorrected *P*-distances and carried out 1000 bootstrap replicates to estimate nodal support. Nodal support values (>75%) were displayed.

## 3. Results

### 3.1. Characterization of Alleles

We successfully and selectively amplified MHC class I exon 3 genes across 80 individuals from four species of the *Turdidae* family using HN36 and HN46 primers. An average of 22.7 clones per individual was sequenced. Sequences varied between 459 and 579 base pairs. The multiple sequence alignments of all sampled species were 411 base pair long. The final aligned MHC class I dataset included 285-291 bp (Primers not include). Analysis of gDNA alignment revealed a total of 77 distinct Haplotypes/alleles including 47 PFA. Each sequence was confirmed to exhibit similarity (81%-93%) with earlier reported passerine MHC class 1 sequences based upon BLAST search. The numbers of PFA sequences found in a single individual ranged from one to five, indicating that one to three loci exist in three of the four species of the *Turdidae* family. However, the number of putative functional alleles found in a single individual ranged from two to seven in *Turdus atrogularis* exhibiting two to four loci. Number of the individual tested, number of PFA and pseudogene retrieved, the minimum number of functional loci estimated is given in Table 1. Three alleles (*Tuna-MHCI*∗*PFA*05 = *Tuen-MHCI*∗*PFA*09, *Tuna-MHCI*∗*PFA*07 = *Tuen-MHCI*∗*PFA*02 and *Tuen-MHCI*∗*PFA*05 = *Tuna-MHCI*∗*PFA*015) were shared among *Turdus naumanni* and *Turdus eunomus.* Two alleles (*Turu-MHCI*∗*PFA*05 = *Tuat-MHCI*∗*PFA*02 and *Turu-MHCI*∗*PFA*09 = *Tuat-MHCI*∗*PFA*08) were also detected among individuals of *Turdus ruficollis* and *Turdus atrogularis.* Interestingly, genotypes comprising of one allele were by far the most repeated (26.67%, 8/30), followed by genotypes comprising two (16.67%, 5/30) and four alleles (13.3%, 4/30) in the population of *Turdus naumanni*. Almost pattern was consistent in population of *Turdus eunomus* and *Turdus rufficollis*. Genotypes constituting one allele (23.3%, 7/30) were the most repeated followed by three (16.67%, 5/30) in *Turdus eunomus.* Genotypes comprising one allele (33.33%, 5/15) were repeated in the population of *Turdus rufficollis.* Allelic repetition was absent in population of *Turdus atrogularis*.

Of the 77 sequences, 30 were non-translatable due to indels or the presence of stop codons resulted changes in the reading frame. Sequences were thus presumed to be pseudogenes. The number of identified pseudogenes within the four species ranged between three and five in most individuals of study population, and six of the thirteen pseudogene sequences were found to be identical in three individuals from the population of sampled species. We cannot ignore the likelihood that some of the identified pseudogene sequences may be due to PCR or sequencing artifacts, as such events would more often result in nonfunctional sequences. The nucleotide deletion result in loss of 3 amino acids was obvious in *Tuna-MHCI*∗*PS*07-9 and *Tueu-MHCI*∗*PS*01-04 and *Tueu-MHCI*∗*PS*08. Both nucleotide deletion, frame shift mutation and premature stop codons were detected in *Turu-MHCI*∗*PS*01,03 and *MHCI*∗*PS*09 at amino acid 33 encoding Exon 3. Loss of 3 amino acids was at position 78 was detected in *Tuat-MHCI*∗*PS*05 and *Tuat-*MHCI∗PS06.

### 3.2. Analysis of Genetic Diversity

Overall we find an elevated genetic diversity (*π*) within exon 3 alleles repertoire among individuals of *Turdus atrogularis* was (0.151) than *Turdus eunomus* (0.113). The average number of nucleotides difference (*K*) varied between 43.95 in *Turdus atrogularis* and 32.32 in *Turdus eunomus.*

### 3.3. Analysis of Recombination

The recombination detection program not only analyzes brake points but also identify parent sequences. We ran the test of recombination by pooling all putative functional alleles recovered from four species of the *Turdidae* family. We only find one potential recombination event in *Tuna-MHCI*∗*PFA06* in *Turdus naumanni* at two recombinant breakpoints at position 148 and 253. *Tuna-MHCI*∗*PFA02* as major and *Tuna-MHCI*∗*PFA011* minor parent. Likewise, a single recombination was significant in *Tueu-MHCI*∗*PFA07.* We detected no recombination among other alleles. However, these recombinations were only significant in two out of seven tests and not consistent with recombination breakpoint identified by GARD, hence the results represent that overall recombination is not likely to have any prominent effects on tests for positively selected sites (Table 2). The recombination breakpoints identified by these two programs are often inconsistent, probably because they use different computational methods.

### 3.4. Analysis of Selection

Considering that the evolutionary history of each domain might have been different, we tested each domain separately for evidence of positive selection. Selection statistics by traditional methods did not disclose any statistical significant signal of selection that deviate from neutral expectations for *Turdus eunomus* (Tajima's *D*: -0.87309, *P* > 0.10; Fu and Li's *D*∗ test statistic: 0.36, *P* > 0.10; Fu and Li's *F*∗ test statistic: 0.03, *P* > 0.10) and *Turdus atrogularis* (Tajima's *D*: -0.86107, *P* > 0.10 Fu and Li's *D*∗ test statistic: 0.19, *P* > 0.10; Fu and Li's *F*∗ test statistic: -0.077, *P* > 0.10). Still, overall *d*_N_ value was significantly higher statistically than *d*_S_ in *Turdus atrogularis (*1.687) and ratio *d*_N_/*d*_S_ was more pronounced at codons presumably coding PBR (1.994) than codons not involved in such activity (0.884) is presented in (Table 3).

Application of Likelihood models represents that the model M8 allows for positive selection provides a better than the neutral evolution models M7. Sites being positively selected were recognized, are given in (Table 4). In total, we find 12 codons under positive selection in sampled species, of which three sites (25%) match homologues codons found positively selected in other avian species and one (8.3%) matched human peptide binding region (Table 4).

**Figure 2:**
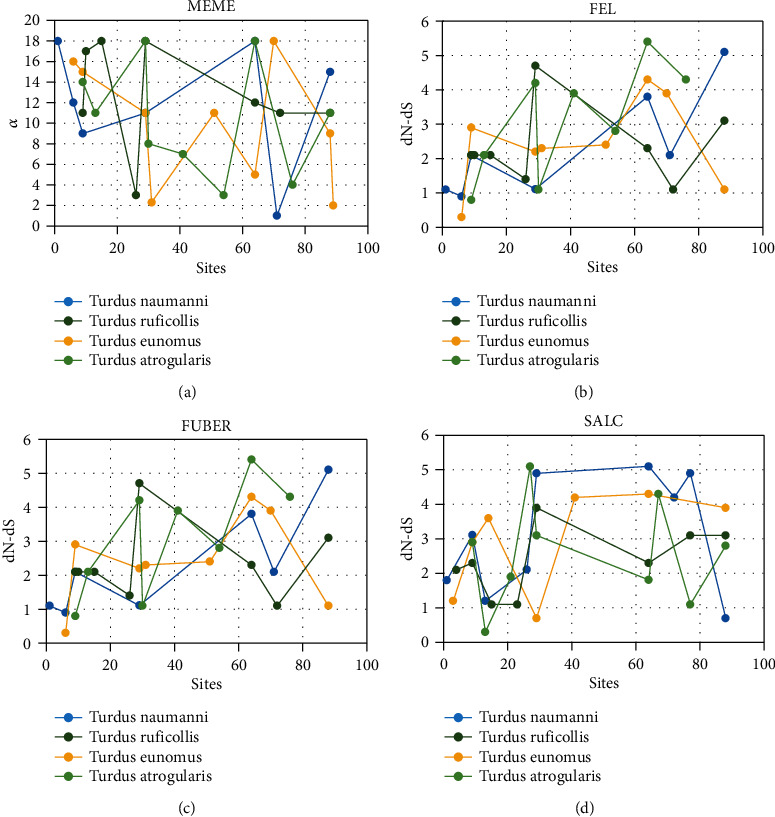
Positively selected sites using the online adoptive evolutionary server Datamonkey with (a) MEME, (b) FEL, (c) FUBAR, and (d) SALC. Substitution tests identify numerous codons showing the signature of positive selection.

**Figure 3:**
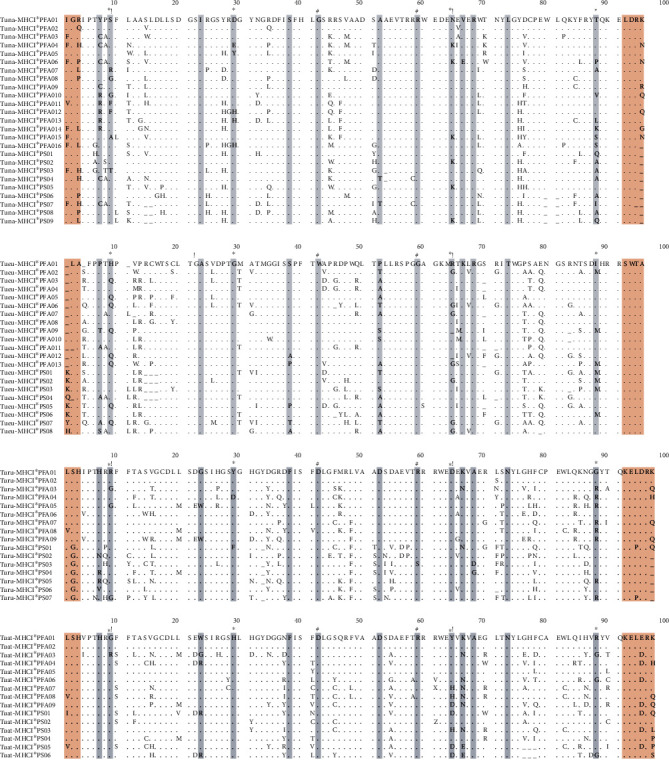
Alignment of deduced amino acid sequences of all alleles retrieved from four species of the *Turdidae* family. Dots (.) indicate identity with the reference sequence. Light gray color represents codons presumably coding for peptide binding regions upon alignment with human and other avian species. The light brown region indicates flanking introns. - indicates missing nucleotides. ∗ represents codons positively selected in almost all of the tests performed for selection analysis. **!** indicates the homologue region also positively selected in most of avian species. **#** inferred homology with human.

Usually consistent with the above finding, every substitute test (MEME, SALC, FEL, and FUBAR) for positive selection implemented in online adoptive evolutionary server Datamonkey (Weaver et al., 2018) identify numerous codons under positive selection (Figure 2) and (Figure 3).

**Figure 4:**
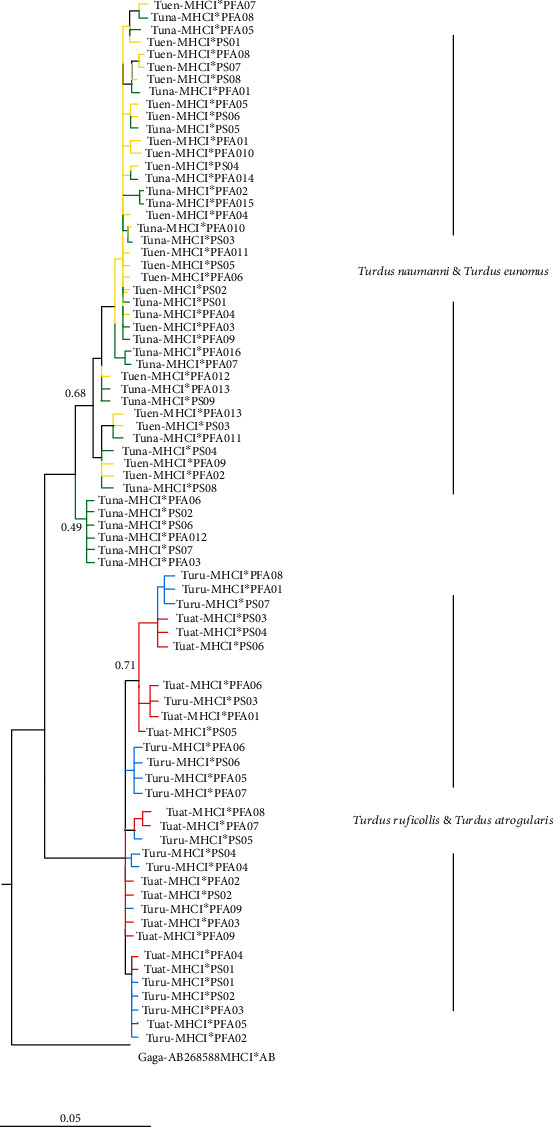
Bayesian phylogenetic reconstruction of MHC class I exon 3 of four species of the *Turdidae* family. All the nodes are well supported (PP > 0.90%) unless indicated otherwise. AB268885 *Gallus gallus* was used as an outgroup.

Across all tests for positive selection, four codons (9, 29, 65, and 88) were frequently identified by all methods as having under positive selection. Of these, codons (42, 59) were corresponding to PBR in human and codons 9, 29, 64, and 88 also match homology to PBR, known as positively selected among passerine in general [56] (Figure 4). The ten most frequent MHC class I alleles retrieved from sampled species displayed 87%-91% sequence similarity to 18 sequences from five other passerine families *(Acrocephalidae, Passeridae, Muscicapidae, Paridae, Passerellidae).* None of the 77 alleles studied had 100% sequence similarity to other published sequences to GenBank; thus, it establishes no allelic pair in the study population that was 100% sequence likeness shared by another species.

### 3.5. Phylogenetic Analysis

In phylogenetic analysis, we observed that sampled species form a well-supported monophyletic clade with *Erithacus rubeculs* members of the *Turdidae* family in maximum likelihood analysis. Bayesian analysis represents that most of the alleles shared among *Turdus atrogularis* and *Turdus reficollis*. This pattern was almost consistent among *Turdus naumanni* and *Turdus eunomus* presented in Figure 4. The Net network of putative functional and pseudogene MHC class I exon 3 sequences in the *Turdidae* family with other passerines indicate that allelic distribution among them is almost congruent with limited divergence. For instance, *Tueu-MHCI*∗*PFA02* and *Tuna-MHCI*∗*PFA07* networks formed a monophyletic clade in the phylogenetic network of exon 3. Three alleles were shared among *Turdus naumanni* and *Turdus eunomus* two among *Turdus rufficollis* and *Turdus atrogularis.* The clustering of the sequences among species could be due to transspecies polymorphism or orthology [57].

## 4. Discussion

In this study, we have for the first time characterize MHC Class I gene in four species of the *Turdidae* family in the order Passeriformes from the wide geographical area of Northeast china. Analysis of MHC class I sequences revealed a total of 77 distinct Haplotypes/alleles including 47 putative functional alleles ever reported in passerine species, a group which is reported to have surprising MHC diversity [58, 59]. According to our findings based on MHC class I sequences, the functional loci in an individual ranged from one to three in three of the four species, which was consistent with findings from other passerine species studied till now [60]. In addition, we detected a large number of presumed pseudogene sequences in the sampled population as it retains important information about the evolution of MHC. This is not surprising, as it is consistent with the expectation of evolution by birth-and-death [61]. We made a significant effort to characterize the variation in regions of MHC class I exon 3 in our study population, we find that the primers would make some unlikely bias in allelic variations among individuals. Hence, MHC class I alleles variations per individual should, largly be due to copy number of genes variation among individuals, which has been confirmed in other birds [62]. Few MHC class I alleles were shared between *Turdus naumanni* and *Turdus eunomus* as well as among individuals of *Turdus ruficollis* and *Turdus atrogularis* is indicating allelic sharing due to common ancestors or challenging common pathogens, as this event is frequent in numerous avian species such as owls, ardeid birds, penguins and passerines [63–65].

Generally, abundant variation in genetic material in a species is an indicator of the capacity to adapt to numerous environmental changes by that species. Rapidly evolving environmental pathogens would cause MHC genes to exhibit enlarged genetic diversity in species [66, 67]. Collectively, in our study, we find elevated genetic diversity among functional sequences and significant divergence, whereas pseudogene has low genetic variation and limited divergence. Similar results also have been described in other passerine species, including common yellow throat [68], great reed warbler and the great tit [69]. The allelic variation described in our study could be due to increased immunological defense against the internal pathogen since these are highly unlikely to adapt to novel, infrequent variant [15].

Recombination has been considered an important mechanism that influences allelic diversity and driving evolution of the MHC gene [70, 71] We only find one potential recombination event in *Tuna-MHCI*∗*PFA06* at two recombinant breakpoints at position 148 and 253 identified with recombination detection program. Similarly, single recombination was significant in *Tueu-MHCI*∗*PFA07.* Recombination pattern was also restricted two out of seven tests; hence our finding indicate recombination is unlikely to have any significant influence on tests for PSs. Though we could not find any substantial recombination among other alleles, qualitatively our result suggests a role for recombination during the evolution of MHC class I in our species studied. Our finding is consistent with, that micro-recombination is frequently observed in MHC genes [57]. Further study of recombinant function in the future will contribute to a detailed understanding of its role in the evolution of the MHC gene.

Positive selection is the maintainer of alleles having the advantageous mutation that maintain fitness of an individual. In our study, the classical test of selection Tajima's *D*, Fu and Li's *D*∗ and Fu and Li's *F*∗ showed no deviation from neutral selection or balance selection. Considering the level of variation, conventional methods used to find selection are not influential [72]. As sites positively selected are likely to accumulate more non-synonymous than synonymous substitutions, influencing amino acid variation to result in functional modifications in proteins [73]. Our study revealed differential expression of selection pattern in functional sequences on regions related with PBR and non-PBR of the MHC class I gene. Codons involved in peptide binding region revealed more non-synonymous substitution than synonymous (*d*_N_/*d*_S_ = 1.99) in *Turdus atrogularis* as compared to non- peptide binding region (*d*_N_/*d*_S_ = 0.884), pattern was consistent among all species tested, which might be enlightened that stronger selection pressure from intracellular pathogens than extracellular pathogens [74]. Evidence of positive selection at PBR of MHC has been reported in the house sparrow (PBR *d*_N_/*d*_S_ = 1.55 vs. non-PBR *d*_N_/*d*_S_ = 0.51) [75] and golden pheasant (PBR *d*_N_/*d*_S_ = 1.45 vs. non-PBR *d*_N_/*d*_S_ = 0.91) [76]. Of the 12 codons in total among species tested exhibit positive selection with Likelihood methods using PAML, 9, 29, 64, and 88 match homologues codons found positively selected in other passerine species.

It should be noted that the pooling of all alleles across loci will mostly reduce selection detection tests, so the outcomes might be conservative, but will be less prone to false positives [77, 78]. Therefore, attention should be given while inferencing about the detected diversity in MHC and the possible effects of selection on individual loci. Our results suggested that *α*2 domain of MHC class I exon 3 of all species are under positive selection pressure. Pronounced positive selection at antigen-binding sites permits a species or population to present a larger repertoire of peptides (antigens), thus increase the defensive ability against parasitic and pathogenic infections.

**Figure 5:**
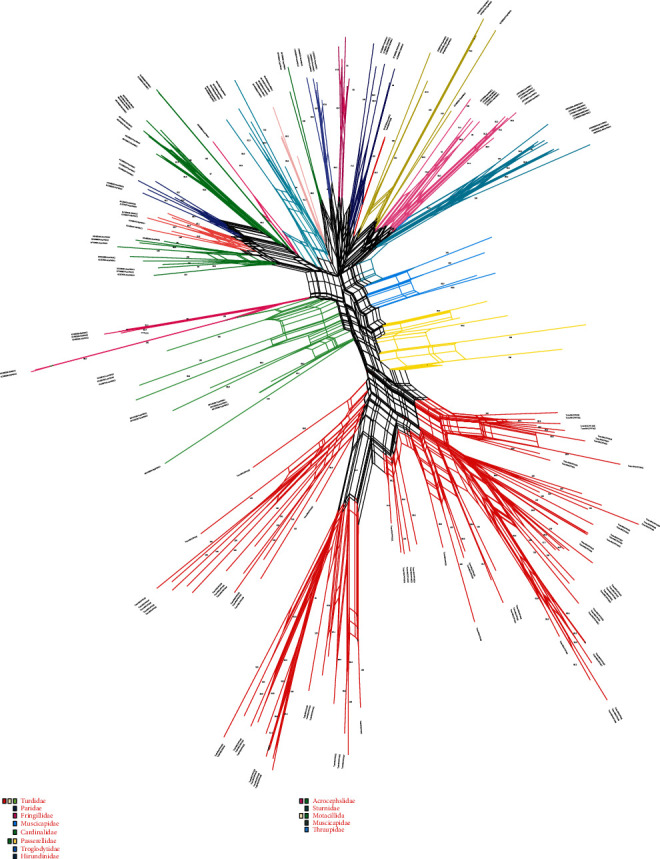
The phylogenetic networks of MHC class I of four species of the *Turdidae* family along with homologue sequences from passerine species. GenBank accession numbers are provided. Species names are mentioned in the lower right side with branch colors. Neighbor-Net networks based on uncorrected *P*-distances and carried out in 1000 bootstrap replicates to estimate nodal support. Nodal support values (>75%) were displayed.

Finally, phylogenetic clustering of MHC class I data set of sampled species when pooled with other passerine species produces a contrasting pattern. In general, the MHC class I sequence of the *Turdidae* family clustered together with sequences from congeneric species. We found increased sequences similarities between the same species rather than within species (trans specific likenesses), is usually described with trans species polymorphism (TSP), which occurs due to alleles passage from ancestral to the decedent via partial arrangement of lineages [79]. Although trans specific similarities can be described with convergent evolution due to the results of similar environmental selective pressure. Studies indicate that TSP is a primary mechanism responsible for clustering of alleles at avian MHC class I [80] (Figure 5).

## 5. Conclusion

Our study shows that species of the *Turdidae* family has retained significant MHC class I diversity, which supports high conservational value and contributes to the evolution of MHC class I genes. Importantly, we specifically amplify the exon 3 locus and provide an opportunity to avoid chimera formation during molecular characterization of hypervariable genes of immunity. At the same time, our study is the first to validate contrasting patterns of allelic diversity and positive selection upon inferred PBR and non-PBR codons which supported the hypothesis that different mechanisms can shape evolutionary paths of MHC class I.

## Figures and Tables

**Table 1:** Amplification success and genetic diversity within each of the four species of the *Turdidae* family investigated. MHC class I exon 3 gene size (*L*), the overall number of polymorphic sites per allele repertoire (*S*), and the average number of nucleotide differences (*K*). Nucleotide diversity *π* at all sites: PBR and non-PBR.

Species	*L*	*S*	*K*	*π*
*Turdus naumanni*				0.118
	285	64	33.7	0.211
				0.091
				0.113

*Turdus eunomus*	285	58	32.32	0.183
				0.079

*Turdus ruficollis*				0.121
	291	65	35.28	0.247
				0.087

*Turdus atrogularis*				0.151
	291	70	43.95	0.309
				0.093

**Table 2:** Recombinants detected in *Turdidae* family MHC class I alleles, parent sequences and breakpoints detected by the recombination detection program (RDP) and the genetic algorithm for recombination detection (GARD), and the RDP analyses.

	Recombination event 1	Recombination event 2
Recombinant	*Tuna_MHCI*∗*PFA06*	*Tuna_MHCI*∗*PFA02*
Maj P	*Tuna_MHCI*∗*PFA02*	*Tuna_MHCI*∗*PFA09*
Min P	*Tuna_MHCI*∗*PFA011*	Unknown (*Tuna_MHCI*∗*PFA01*)
BP 1 location	148 (148)	254 (253); *P* < 0.001
BP 2 location	Absent	Absent

RDP methods		
RDP	NS	<0.05
GENECONV	<0.001	<0.001
BootScan	<0.01	<0.05
MaxChi	<0.001	<0.01
Chimaera	<0.01	<0.01
SiScan	<0.001	<0.001
3Seq	<0.001	<0.01

BP from GARD	Absent	

Note: NS indicates not significant. Maj *P* and Min *P* represent major and minor parents, respectively. BP denotes breakpoint. The numbers in parentheses are BP locations in the recombinant nucleotide sequences without gaps. The values after the semicolon are Max chi values for those BPs. ∗∗ indicates *P* < 0.01.

**Table 3:** The average rates of nonsynonymous (*d*_N_) and synonymous (*d*_S_) substitutions and the result of *Z*-test and the average nucleotide distances (*d*_nt_) and amino acid distances (*d*_aa_) for PBR and non-PBR and all sites in MHC class I of the *Turdidae* family.

Species	Domain	*d*_N_ ± SE	*d*_S_ ± SE	*Z*	*P*	*ω*	*d*_nt_ ± SE	*d*_aa_ ± SE
*Turdus naumanni*	All sites	0.142 ± 0.026	0.104 ± 0.013	0.610	0.543	1.365	0.051 ± 0.08	0.246 ± 0.035
	PBR	0.281 ± 0.059	0.153 ± 0.032	1.848	**0.034**	1.835	0.041 ± 0.045	0.372 ± 0.082
	Non-PBR	0.051 ± 0.062	0.067 ± 0.012	1.356	0.476	0.761	0.034 ± 0.09	0.126 ± 0.029

*Turdus eunomus*	All sites	0.134 ± 0.021	0.107 ± 0.019	0.729	0.457	1.251	0.043 ± 0.010	0.202 ± 0.031
	PBR	0.179 ± 0.023	0.102 ± 0.032	1.442	0.383	1.175	0.057 ± 0.031	0.366 ± 0.011
	Non-PBR	0.056 ± 0.041	0.049 ± 0.012	1.792	1.000	1.142	0.051 ± 0.011	0.134 ± 0.029

*Turdus ruficollis*	All sites	0.146 ± 0.048	0.101 ± 0.025	0.911	0.771	1.445	0.059 ± 0.017	0.191 ± 0.035
	PBR	0.264 ± 0.037	0.138 ± 0.011	1.643	**0.002**	1.912	0.147 ± 0.045	0.453 ± 0.078
	Non-PBR	0.070 ± 0.018	0.068 ± 0.028	0.061	0.476	1.029	0.034 ± 0.049	0.126 ± 0.052

*Turdus atrogularis*	All sites	0.189 ± 0.091	0.112 ± 0.025	1.040	0.150	1.687	0.063 ± 0.053	0.211 ± 0.039
	PBR	0.321 ± 0.013	0.161 ± 0.069	1.012	0.435	1.994	0.207 ± 0.045	0.572 ± 0.162
	Non-PBR	0.069 ± 0.062	0.078 ± 0.012	1.813	0.476	0.884	0.078 ± 0.091	0.206 ± 0.041

^∗^The errors were attained through 1000 bootstrap replicates which are in parentheses. Bold represents significant results.

**Table 4:** Estimation of *d*_N_ and *d*_S_ substitution rates for sites positively selected and their ratio for codons chosen a priori (PBR and non-PBR).

Species	Comparison	Model	lnL value	Parameter estimates	PSSs	LRT	TS value
*Turdus naumanni*	Tuna 1-30	M7 (beta)	-703.53	*P* = 0.19, *q* = 0.138, *P*_0_ = 0.351	Not allowed	M7 vs. M8	5.47
		M8 (beta and omega)	-722.49	*P* = 0.87, *q* = 0.117, *P*_1_ = 0.09, *ω* = 3.11	39F,41L 88T		

*Turdus eunomus*	Tuna 1-30	M7 (beta)	-692.13	*P* = 0.13, *q* = 0.97, *P*_0_ = 0.347	Not allowed	M7 vs. M8	4.56
		M8 (beta and omega)	-714.11	*P* = 0.91, *q* = 0.158, *P*_1_ = 0.11, *ω* = 3.76	41L, 52P,88T		

*Turdus ruficollis*	Turu1-15	M7 (beta)	-811.27	*P* = 0.49, *q* = 0.187, *P*_0_ = 0.411	Not allowed	M7 vs. M8	6.21
		M8 (beta and omega)	-834.88	*P* = 0.131, *q* = 0.232, *P*_1_ = 0.13, *ω* = 3.94	29Y, **78F**, 88G		

*Turdus atrogularis*	Tuat1-5	M7 (beta)	-847.53	*P* = 0.83, *q* = 0.211, *P*_0_ = 0.585	Not allowed	M7 vs. M8	7.10
		M8 (beta and omega)	-849.78	*P* = 0.173, *q* = 0.279, *P*_1_ = 0.17, *ω* = 4.11	39H, 78F, **91Q**		

^∗^The log likelihood values and parameters estimated were computed using codeml implemented in PAML 4.9. PSSs were inferred in model M8 by BEB with posterior probabilities > 95%.

## Data Availability

The data of this study will be available openly to readers, and they can access the data supporting the conclusions of the study.

## Abbreviations

MHC: Major histocompatibility complex
PBR: Peptide-binding region
CDs: Cluster of differentiation
CDRs: Complementary-determining regions
ABS: Antigen-binding site
TCR: T-cell receptors
SDS: Sodium dodocyl sulfate
EDTA: Ethylene diamine tetra acetic acid
PCR: Polymerase chain reaction
HLA: Human leukocyte antigen
MEGA: Molecular Evolutionary Genetics Analysis
GTR: General time-reversible model
PFA: Putative function alleles
GARD: Genetic algorithm for recombination detection
RDP: Recombination detection program
PP: Posterior probability
BP: Breakpoint
*d*_N_: Nonsynonymous substitution
*d*_S_: Synonymous substitution
*d*_nt_: Nucleotide distance
*d*_aa_: Amino acid distance
SE: Standard error
PSSs: Positively selected sites
BEB: Bayes empirical Bayes
MEME: Mixed effects model of evolution
SALC: Single likelihood ancestor counting FEL, fixed effect likelihood
FUBAR: Fast unconstrained Bayesian approximation
PAML: Phylogenetic analysis using maximum likelihood
TSP: Transspecies polymorphism.
